# Pediatric oncology healthcare professionals’ attitudes to and awareness of regulations for minors’ and guardians’ online record access: a mixed-methods study in Sweden

**DOI:** 10.1186/s12913-025-13697-3

**Published:** 2025-11-27

**Authors:** Josefin Hagström, Charlotte Blease, Arja Harila, Isabella Scandurra, Päivi Lähteenmäki, Maria Hägglund

**Affiliations:** 1 Participatory eHealth and Health Data Research Group, Department of Women’s and Children’s Health, Uppsala University, Uppsala, Sweden; 2 Digital Psychiatry, Department of Psychiatry, Beth Israel Deaconess Medical Center, Boston, MA USA; 3 Pediatric Oncological and Neurological Research, Department of Women’s and Children’s Health, Uppsala University, Uppsala, Sweden; 4 Centre for Empirical Research on Information Systems (CERIS), Informatics, School of Business, Örebro University, Örebro, Sweden; 5 Department of Women’s and Children’s Health, Karolinska Institute, Stockholm, Sweden; 6 Department of Pediatric and Adolescent Medicine, Turku University Hospital, Turku, Finland; 7 Medtech Science & Innovation Centre, Uppsala University Hospital, Uppsala, Sweden

**Keywords:** Healthcare professionals (HCPs), Oncology, Adolescents, Adolescent health, Patient accessible electronic health record (PAEHR), Electronic health record (EHR), Patient portal, Survey, Ehealth, Interviews

## Abstract

**Background:**

Healthcare providers and policymakers worldwide differ in their provision of access to adolescent patients’ electronic health records (EHR). The regulatory framework in Sweden restricting both guardians’ and adolescents’ online record access (ORA) has during recent years received criticism. The aim was to quantitatively and qualitatively, explore attitudes about ORA and perceptions about ORA regulations among pediatric oncology healthcare professionals (HCPs) in Sweden.

**Methods:**

A convergent mixed-methods design (QUAL, quan) was used, consisting of a survey study (*N* = 95) and semi-structured individual interviews (*N* = 13). Physicians and nurses in pediatric oncology were recruited in clinics face-to-face or via staff e-mail. Descriptive statistics were used to present quantitative survey results. Interviews were recorded, transcribed, and analyzed using content analysis.

**Results:**

A majority of participants (72%) were critical of the access restrictions but lacked knowledge about access extensions, with more than 60% unaware of application procedures. Five themes emerged regarding both perceived benefits and risks of ORA. Examples of benefits included adolescent empowerment, parental support, and improved partnership; risks included an increased emotional distress and confusion among young patients and their guardians, increased workload for HCPs, and threats to adolescent confidentiality. An additional five identified themes captured HCPs’ views on regulations and included uncertainty, variation among adolescents, and the need to balance parental support and adolescent privacy.

**Conclusions:**

Findings indicate lacking knowledge about ORA regulations and little incentive for HCPs to promote its use. While risks of ORA were often directly experienced and concerned confidentiality breaches and difficulties with EHR documentation, benefits tended to be anticipatory and related to patient or parent experiences. Still, HCPs showed limited support for ORA restrictions during adolescence. To ensure safe and effective ORA use, HCPs need clearer guidance and support.

**Trial registration:**

Not applicable.

**Supplementary Information:**

The online version contains supplementary material available at 10.1186/s12913-025-13697-3.

## Introduction

Electronic health records (EHRs) are essential to function as an aide memoir and communication tool among healthcare professionals (HCPs) [1]. Currently, providing patients and caregivers with online record access (ORA) via patient portals is becoming increasingly common among healthcare providers internationally. In the European Union (EU), the General Data Protection Regulation (GDPR) provides individuals with the right to check the data about them in registries such as EHRs. A proposal for a European Health Data Space has been launched, which will give patients online access to their EHRs throughout Europe. A growing body of research recognizes unique benefits of ORA among adolescents and parent proxy users, including adolescents’ supported transition into adult care and improved parental care [2, 3]. However, ORA in pediatric care remains a controversial topic and scarce attention has been paid to the views and experiences of portal access policies among HCPs working with seriously ill children.

Studies over the last two decades have identified initial provider reluctance to patient ORA that tends to diminish yet prevail to an extent after implementation [4, 5]. Some HCPs perceive patients’ access as interfering with the function of the medical record as their work tool [6], and state that it can contribute to HCP burnout [7]. Pediatric oncology HCPs’ views and experiences of ORA have been addressed in one US study, where interviewed physicians and advanced practice providers reported benefits in empowering adolescents and supporting parental care. While anticipating that parents losing access might lead to great inconvenience, they worried about adolescents and parents accessing bad news in the EHR without explanation, and reported changing their documentation as a result [8]. HCPs’ concerns around ORA may be amplified by the severity and complexity of childhood cancer, which often involves distressing information in the EHR and emotional vulnerable patients and families. In fact, oncologists are less likely than non-oncologists to see ORA as a way to improve patient safety, less likely to believe patients will take better care as a result, and more likely to think patients will feel in control [9]. Oncologists in particular are concerned about how sharing notes with patients will impact documentation practices [9, 10].

In pediatrics, an additional and persisting concern is patient confidentiality breaches and maintaining the privacy of minors’ information, particularly during adolescence [11–13]. A scoping review identified confidentiality as the main concern among pediatric HCPs and experts [14]. The confidentiality issue stems from the challenging balance between parental responsibility and adolescents’ increasing desire for independence. While parents are often given full access to their child’s records during early childhood, adolescents may desire increased autonomy and privacy. One of the barriers to providing confidential care is limited portal functionality [15, 16]. where HCPs are not equipped with tools to conceal information from adolescents and parents in a satisfactory way [17].

One strategy aimed at resolving this issue has been to limit adolescents’ and parents’ access during adolescence through regulations. Yet, implementation of ORA for parents and adolescents differs globally, in terms of allowance of parental access and the age when the patients can access their records on their own [18, 19]. A variety of access control practices attempting to balance parents’ and adolescents’ needs have been adopted, where practices are either based on set age-based access limits or on case-by-case assessment.

### Study aim

Uncertainty remains about HCPs’ views on existing regulations that concern adolescents and parents. Though ORA is an evolving innovation, national patient portals have been accessible for citizens in Sweden and other Nordic countries for more than a decade. The study aimed to investigate, both quantitatively and qualitatively, Swedish pediatric oncology HCPs’ perceived benefits and risks of adolescents’ and parents’ ORA, as well as their views on and awareness of ORA regulations. The study addresses two research questions:

#### RQ1

What benefits and risks do Swedish pediatric oncology HCPs perceive regarding adolescents’ and parents’ ORA?

#### RQ2

What are Swedish pediatric oncology HCPs’ awareness of and views on the national regulatory framework around parental and adolescent access to minors’ online records?

## Methods

### Study design

A convergent QUAL-quan mixed-methods approach was adopted based on a belief that quantitative and qualitative data would complement each other [20, 21]. Mixed-methods can be defined as “collection of both qualitative and quantitative methods where data is integrated in the analysis” [22]. Because the topic has not previously been studied, a survey was used to provide a breadth of data, and interviews were performed to enable a deeper comprehension of the underlying reasoning behind the quantitative findings. For example, survey data on HCPs’ views on ORA regulations were complemented with interview insights to develop a more nuanced understanding of their concerns. In this way, the integration of both data types enabled us to cross-validate and further interpret the results. Data collection occurred from March 2022 to May 2023 after ethical approval from the Regional Ethical Review Board in Uppsala, Sweden (EPN 2022/02160). The qualitative component is reported according to the Consolidated criteria for Reporting Qualitative research (COREQ) guidelines, see Supplementary Material 1 [23].

### Participants and setting

Purposive sampling was used. Participants were recruited via e-mail (sent from administrators to staff) at hospitals and oncology healthcare organizations in 13 of 21 Swedish regions, and information in person at Uppsala University Hospital in Uppsala, Sweden. Initially, HCPs were recruited in person and via e-mail at Uppsala University Hospital. Due to low survey participation, two pediatric oncology HCPs on the research team (AH and PL) assisted by identifying and reaching out to colleagues in other regions. Surveys were distributed via administrative staff in various clinics across 13 regions, and reminders were sent on one occasion through the same channels. We aimed to achieve broad variation by inviting HCPs from multiple regions and professional backgrounds to reflect different experiences with ORA.

HCPs were included who had experience with working with children and adolescents with cancer and who had experience of documenting in the EHRs. Consent was provided by completing the survey. At the end of the survey, participants were able to register interest in participating in an interview and provide their e-mail and/or phone number. Contact information was stored separately from the survey results, ensuring anonymity of the survey. While no incentives were offered for completing the survey, interview participation was rewarded with a gift card of 200 SEK (equivalent to approx. €20).

In Sweden, where around 300 children are diagnosed with cancer each year [24], ORA is advanced and adolescents and parents have access to the PAEHR service 1177 journal via the national patient portal 1177.se. Parents (or legal guardians) have automatic access to their child’s record from birth until they turn 13, and at 16 years old, the child themselves gain access. In the years between 13 and 16, neither parent nor child have access to the record. By filling in and submitting a paper form for an assessment by the operations manager, parents can be granted prolonged access or teenagers/children can be granted earlier access. The portal access policy is not based on Swedish law but on common practice within healthcare [25], and was set in 2017 by the company managing the web platform [26]. The policy has been criticized by parents of ill children, such as cases of cancer [27] or severe disability [28]. Since before PAEHR implementation, patients and parents have been able to request a physical record copy, which necessitates an individual assessment by the provider whereby the request can be denied. There are also ‘hidden search words’ that can be used by HCPs to conceal information from patients’ view, for example “Early hypotheses”, “Intimate partner violence”, and “Report of concern for a child’s welfare”. In Sweden, specialist care is delivered in six university hospitals only, but the shared care model applies and, thus, all regions do have some share in the follow-up of the treatment. In Sweden, specialist care is delivered in six university hospitals only, but the shared care model applies and, thus, all regions do have some share in the follow-up of the treatment.

### Data collection

#### Survey

**Fig. 1:**
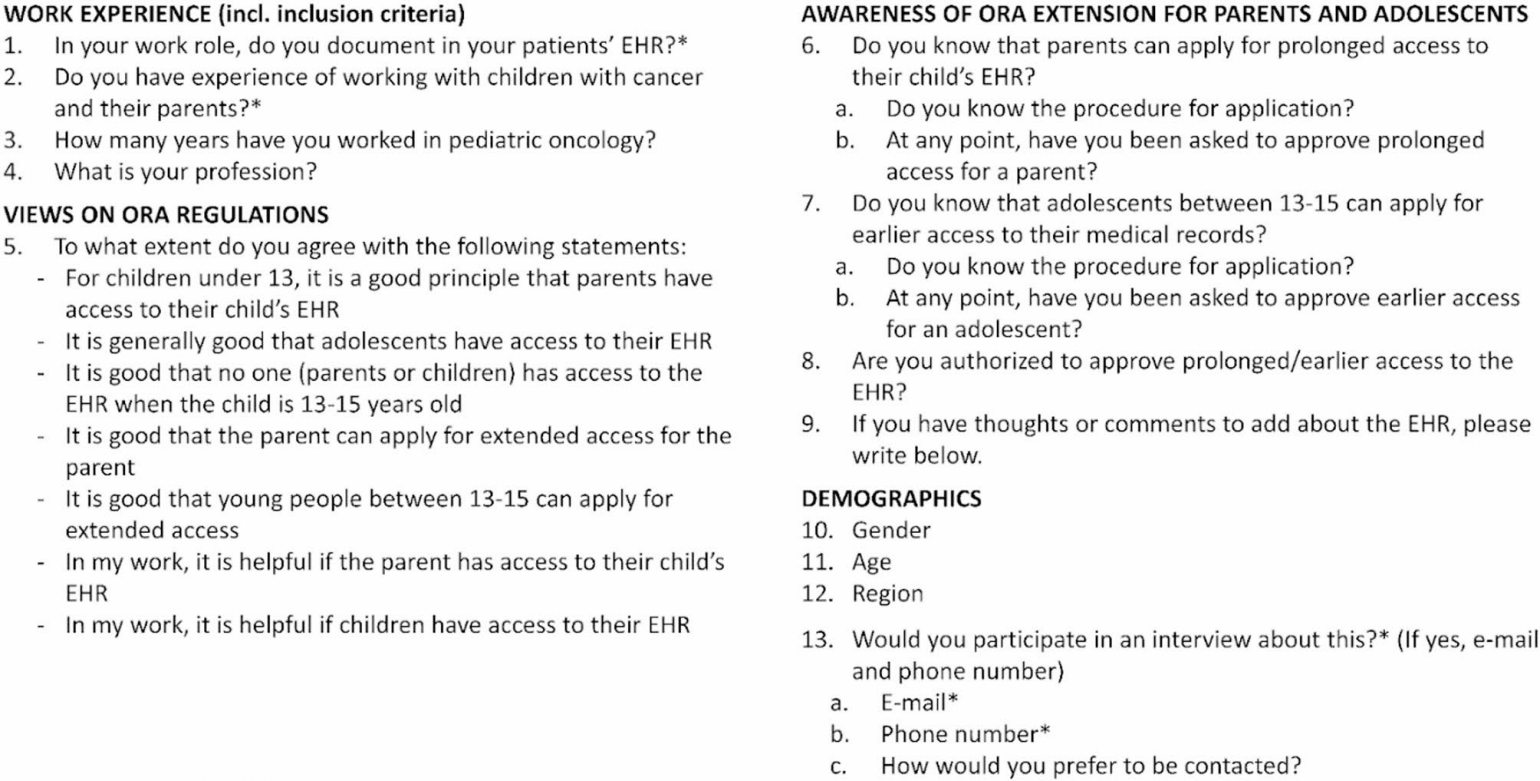
Survey questions included in the study

A survey instrument of 20 questions was developed in Swedish. by the authors, and piloted with a small group of pediatric healthcare professionals to refine wording and usability before full deployment. The survey questions were directly mapped to the research aims by developing themes that reflected key aspects of HCPs’ experiences with ORA, such as their views on and awareness of access ages and perceptions of its impact. For this study, 13 questions were included based on the aim: four questions regarding experience, one on views on regulations, three on awareness (with four conditional questions), three on demographics and one on interview participation, see Fig. 1. Questions were optional, except for those related to inclusion criteria and contact information, which were required if the participant expressed interest in an interview. The online survey was conducted using REDCap (Vanderbilt). The full survey in Swedish and English can be found in Supplementary Material 2.

#### Interviews

The first author conducted the interviews between February 2022 and April 2023 via telephone or video-conferencing software. An interview guide was created based on prior work on ORA for adolescents, children, and parents (see Supplementary Material 3) and included similar themes as surveys. The interview guide was revised based on input from a stakeholder advisory board and 3 informatics administrators. Interviews were audio-recorded and professionally transcribed. JH is a PhD student in health informatics with experience of conducting studies using qualitative analysis and several completed courses in qualitative research. JH had no prior relationship with any of the study participants. Data were collected until saturation was reached, as determined by JH, who conducted all interviews and reviewed the data throughout the collection process.

### Data analysis

Descriptive statistics were used to present quantitative survey data. All but one of interviews were transcribed by an expert, and one was transcribed by the main author. A thematic analysis with an inductive approach was conducted by JH and MH using the software program NVivo (www.qsrinternational.com/nvivo-qualitative-data-analysis-software/home). MH is a researcher in health informatics and ORA implementation, with extensive experience in qualitative analysis of interviews. As views on regulations were previously unexplored, we analyzed this interview data using the six steps of Braun and Clarke [29]: (1) authors read transcripts to familiarize themselves with the data, (2) data was categorized into codes, (3) codes were grouped into categories and themes, and (4) definitions were refined further through discussions during meetings. Any discrepancies were resolved through discussion until consensus was reached. Discussions of the findings with all authors led to refinement of the themes. Analysis of perceived benefits and risks was inspired by previous work [3, 8]. Quantitative and qualitative results were presented separately but integrated through triangulation during the interpretation phase. Themes from the qualitative data were compared with survey findings to identify points of agreement and difference, providing a fuller understanding of HCPs’ experiences with ORA.

## Results

### Sample characteristics

Of 124 HCPs who responded to the survey, 95 (77%) completed the survey and were included in the study. Eight (7%) were excluded because they did not meet the inclusion criteria and 21 did not complete the survey (17%). Most participants were women (83/95, 88%) and worked as nurses (66/95, 70%). 17 selected their profession as ‘other’, citing physiotherapist, child specialist, specialist nurse, pediatric nurse, unit manager, and assistant nurse. HCPs from eight regions responded to the survey. Demographic characteristics of those interviewed, those who responded to the survey but did not partake in interviews, and all participants (survey respondents who were and were not interviewed) are shown in Table 1.

**Table 1:** Survey and interview participants’ demographic characteristics and work experience

Characteristic	HCP		
	Interviewed (*n* = 13)	Not interviewed (*n* = 82)	All (*N* = 95)
**Gender**,** n (%)**			
Man	3 (23.1)	8 (9.8)	11 (11.6)
Woman	10 (76.9)	74 (90.2)	84 (88.4)
Other	0 (0)	0 (0)	0 (0)
**Age**,** n (%)**			
18–24 years	1 (7.7)	5 (6.3)	6 (6.3)
25–34 years	3 (23.1)	23 (29.1)	26 (27.4)
35–44 years	2 (15.4)	19 (24.1)	21 (22.1)
45–54 years	5 (38.5)	12 (15.2)	17 (17.9)
55–64 years	1 (7.7)	20 (25.3)	21 (22.1)
65 years or older	1 (7.7)	0 (0)	1 (1.1)
Missing	0 (0)	3 (5.7)	3 (3.2)
**Years of practice**,** n (%)**			
One year or less	1 (7.7)	6 (7.3)	7 (7.4)
2–5 years	2 (15.4)	27 (32.9)	29 (30.5)
6–10 years	0 (0.0)	14 (17.1)	14 (14.7)
11–15 years	4 (30.8)	10 (12.2)	14 (14.7)
16–20 years	2 (15.4)	8 (9.8)	10 (10.5)
20 years or more	4 (30.8)	17 (20.7)	21 (22.1)
Missing	0 (0)	0 (0)	0 (0)
**Profession**,** n (%)**			
Physician	4 (30.8)	18 (22.0)	22 (23.2)
Nurse	8 (61.5)	58 (70.7)	66 (69.5)
Other	1 (7.7)	6 (7.3)	7 (7.4)

Of 95 HCPs who completed the survey, 19 (20%) agreed to partake in an individual interview. Subsequently, six participants did not partake in interviews due to scheduling difficulties and lack of response, leaving 13 (68.4%) interviewees. A majority of interview participants (8/13, 61.5%) were female, mean age was 45 years old, and mean number of years of experience was 15 years. Interviews (ranging between 20 and 79 min) were audio-recorded and all but one were transcribed by a professional (one was transcribed by JH). Interview participants’ characteristics are shown in Table 2.

**Table 2:** Interview participants’ characteristics

ID	Gender	Age	Profession	Years of clinical experience in pediatric oncology	Interview setting
02	Male	45–54	Nurse	1–5	Video
03	Female	55–65	Nurse	21–25	Video
06	Female	18–24	Nurse	1–5	Video
18	Female	35–44	Physician	11–15	Phone
22	Female	55–64	Nurse	31–35	Video
33	Female	35–44	Nurse	11–15	Video
41	Female	45–54	Physician	11–15	Video
69	Female	25–34	Nurse	6–10	Phone
72	Male	45–54	Physician	11–15	Video
81	Male	25–34	Nurse	1–5	Video
104	Female	45–54	Physiotherapist	11–15	Video
116	Male	65+	Physician	35–40	Video
118	Female	34–44	Nurse	11–15	Video

### Quantitative results

**Fig. 2:**
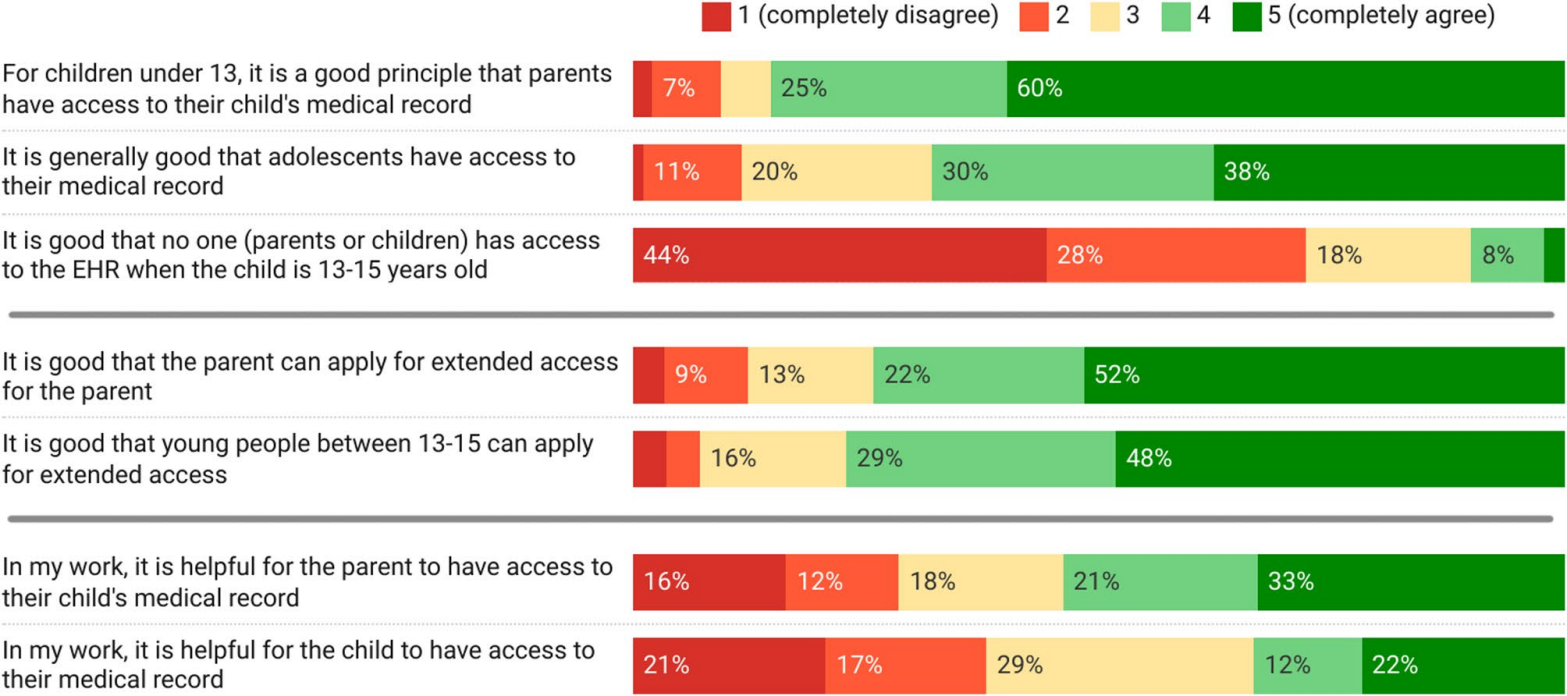
HCPs’ views on parental and adolescents’ online record access

Very few (10%) reported the view that the gap in ORA for parents and adolescents during the period between the ages of 13 to 15 was a good thing. Meanwhile, a larger proportion of HCPs (81/95, 85%) were positive about parents’ access for children under 13 than to adolescents’ access (63/93, 68%), see Fig. 2. About three fourths of HCPs were positive about the option for adolescents (64/83, 77%) and parents (66/89, 74%) to apply for extended access outside of the default. While more than half (49/91, 54%) considered parental access to the adolescent’s records as useful for their work, HCPs diverged in their responses regarding the utility of adolescents’ access, as 29% (25/87) selected the middle option and 38% (33/87) did not agree. See detailed results in Supplementary Material 4.

Content in free-text comments were mirrored in interviews, except for one comment stating that medication management became a big issue for parents when losing access.

While 62% (58/94) knew that parents can apply for extended access, three out of five (34/56, 61%) did not know the procedure of application. Even fewer HCPs - only a fifth - were aware that adolescents were able to apply for earlier access (18/94, 19%). Of these, two thirds (12/18, 67%) did not know the procedure for applying. Almost half (26/58, 45%)) of respondents who were aware of the possibility to apply for access extension did not know whether they were authorized to approve such applications.

### Qualitative results

#### Perceived benefits of ORA for adolescents, parents, and HCPs

In general, HCPs’ experiences differed. While one HCP perceived ORA to cause problems to a degree that it should not be offered, another had worked for 40 years and never heard any parent or adolescent mention the EHR despite ORA being implemented for more than 10 years. HCPs reported five themes relating to perceived benefits of adolescent and parental ORA for adolescents, parents, and HCPs; *Improving adolescents’ and parents’ emotional state*,* Facilitated parental care management*,* Empowering adolescents*,* Improved partnership and communication* and *Enhanced documentation accuracy*. For details, see Table 3.

**Table 3:** Themes identified in interviews in regards to hcps’ perceived benefits of adolescent and parental ORA for adolescents, parents, and HCPs

Themes	Representative quote
Improving adolescents’ and parents’ emotional state	“It’s stressful for all parents even if they can read the EHR, but it becomes an increased stress [without it] because then they are on pins and needles, waiting for a doctor to call.” (Participant #03, nurse with 22 years’ experience) “Parents sometimes say, ‘It helps me that I can go in and see the test results myself, then I feel more secure.’ And some parents make Excel sheets, then it’s easier for them to go in themselves, they think it gives them a sense of control in a different way.” (Participant #18, physician with 12 years’ experience)
Facilitated parental care management	“There is chaos in the families that come to us. It is difficult to keep track of everything, ‘when is the next treatment? What was said?’ Both parents may not have been present at the hospital to get the information verbally.” (Participant #02, nurse with 23 years’ experience) “A child experiencing a life crisis when they’re still young and perhaps shouldn’t have to deal with those life crises, I think it’s good that [parents] can be there as a support and be able to access medical records to help them, for example, to remember when they have treatments and so on.” (Participant #81, nurse with 5 years’ experience)
Empowering adolescents	“You can take own initiative and go in and check things if you have questions, instead of accepting it or turn to mom or dad […]. You, yeah, can do some things by yourself.” (Participant #69, nurse with 6 years’ experience) “Sometimes the teens are very competent and are undergoing a sort of continuous therapy where one is not at risk of big surprises, and where they can feel that they can gradually take responsibility for their illness. If you are 17 and a half and maybe are transitioning from child clinic to an adult clinic, it’s good that one has been able to practice.” (Participant #72, physician with 12 years’ experience)
Improved partnership and communication	“We are only two people, so we don’t always answer [the phone] right away, sometimes it’s the answering machine, and it takes a bit of time before we call back, and often they want to know the test results right away when they call the first time. And then it’s quicker for them to just log in and look.” (Participant #33, nurse with 13 years’ experience) “If [parents] haven’t been present at the visit, they’ll know if the child has been prescribed a certain treatment that they’re not aware of.” (Participant #104, physiotherapist with 13 years’ experience)
Enhanced documentation accuracy	“It’s nothing that would cause any problem for us, but rather it’s good because it’s important that it’s accurate. And then you have extra pairs of eyes as well, [parents] only have their child’s record while we have a lot of EHRs, and of course, there’s always a risk of something going wrong.” (Participant #33, nurse with 13 years’ experience) “There can also be errors when [adolescents] say, “this was in the record, this isn’t correct.” And that’s also good because then we get to see what’s misunderstood.” (Participant #118, nurse with 13 years’ experience)

Participants reported that ORA helped in *improving adolescents’ and parents’ emotional state*. For example, they described having immediate access to test results led to an increased sense of control and a sense of safety. Furthermore, it was anticipated that adolescents and parents may feel reassured that they have understood correctly or that agreed-upon procedures have occurred: “In the best case, [parents] feel reassured when they read the EHR and see that the same things they understood verbally. So, it can be very positive to be able to go back after a meeting and see what was planned as well. […] That you can get some confirmation that certain things that were agreed upon have been carried out.” (Participant #72, physician with 12 years’ experience).

Participants reported that ORA led to *facilitated parental care management*, perceiving that the innovation enabled parents to stay involved with their child’s care and provide various types of support. For example, ORA provided busy and worried parents with memory aid for remembering medication and appointments. Reading the record provided an important tool, diminishing the pressure to remember every detail stated during a visit. Participants also mentioned that ORA lessened the burden on the child and allowed parents support their child better, by managing and preparing for appointments.

Several participants described that ORA was *empowering adolescents* to be more involved in their own care. While highlighting that all adolescents did not have the interest to do so, HCPs were positive to having the choice to do so, emphasizing that adolescents had right to their information. Also, their understanding may be improved by not only hearing information, but also reading it afterwards. Participants also foresaw that ORA facilitated a gradual transition into adult healthcare. One nurse stated that having ORA allowed adolescents to reflect on their own: “I don’t think [teens] always care so much about the conversations they have with the doctor in person during the day with their parents present, but I think sometimes they may need to be undisturbed when they want to be.” (Participant #118, nurse with 13 years’ experience).

Some participants reported that ORA enabled *improved partnership and communication*. For example, they saw a benefit in that adolescents and parents could go back and read, to prepare questions. Furthermore, they suggested information in the EHR was more accurate than if adolescents would have googled to find something out on their own. One nurse envisioned that adolescents reading results in their EHR would aid their understanding of why treatment was needed; “Even though [adolescents] are not the ones making decisions, they might have understanding when the healthcare team calls and says, ‘You need to come to the hospital for a blood transfusion.’ If they’ve seen that ‘yes, my hemoglobin was very low when I took the test,’ they don’t need to question it.” (Participant #22, nurse with 34 years’ experience).

Some also contended that ORA enabled parents to stay involved with the child’s care even if unable to attend an appointment. A few HCPs stated that there was potential for using the EHR as a tool to for example, communicate test results to families, however this was hindered by the inability to know or assume that adolescents and parents would in fact check the EHR. Furthermore, they suggested that ORA relieved parents from having to call the clinic to ask about test results. When parents lost access at the adolescent’s age of 13, HCPs reported that parents began calling to request new test results and that, nurses, in particular, needed to print written information.

Participants perceived that ORA contributed to an *enhanced documentation clarity and accuracy*, as it enabled adolescents and parents to notify HCPs of inaccuracies or misunderstandings that they identified in the EHR. Several HCPs emphasized that errors often consisted of parents misunderstanding the EHR or HCPs having misunderstood, rather than what they called an error. One HCP noted: “No one has the intention to write inaccuracies in the record, but it can still turn out wrong for the patient and the family.” [Participant #18, physician with 12 years’ experience].

#### Perceived risks of ORA for adolescents, parents, and HCPs

HCPs reported five themes of perceived risks of adolescent and parental ORA for adolescents, parents, and HCPs; *Increased emotional distress and confusion*,* Decreased documentation quality and accuracy*,* Increased workload*,* Threatened confidentiality*, and *Technical portal limitations*. For details, see Table 4.

**Table 4:** Themes identified in interviews in regards to hcps’ perceived risks of adolescent and parental ORA for adolescents, parents, and HCPs

Theme	Representative quote
Increased emotional distress and confusion	“That’s the case with all patients and not just for children, I think. That it can be a bit scary if you read and google and don’t understand everything.” (Participant #06, nurse with 1 year of experience) “When [HCPs] present the results for examination or things like that, I sometimes think that parents don’t understand that there have been many discussions, many involved parties with opinions who are experts in their fields who have said something.” [Participant #118, nurse with 13 years’ experience] “With cancer, some parents can become very… they read the records several times a day. […] They can notice words, like “they wrote this and they wrote that”, and they lose control.” (Participant #104, physiotherapist with 13 years’ experience)
Decreased documentation quality and accuracy	“The difference now is that one actually has to omit important current information, instead one has to… we have to write on paper notes and try to convey important information between ourselves, which can mean a patient safety risk, that we cannot write things down in the records as one would wish.” (Participant #41, physician with 12 years’ experience) “It can be a bit harder to understand how a nurse… perceived the room. For example, these young children that we admit for food observation, […] we look at least as much at how the parents are doing. Are the parents reacting to the child’s signals, do they seem attached? Are they picking up the child? Like that. And sometimes if you express it… Sometimes it’s much harder to clearly convey what you see in the room if you can’t use any value-loaded words.” (Participant #18, physician with 12 years’ experience)
Increased workload	“[Parents] receive news where we can’t provide support in the same way, and we are noticing now, it creates a lot of extra work for us in healthcare when we have to take care of this anxiety afterwards. Or they call and ask what it means, what it implies, and so on.” (Participant #41, physician with 12 years’ experience) “[Parents] can even sit with their phone while I’m doing my treatment, and comment out loud on what is said in front of the child as well, in front of me and everything. And then they can get upset if something doesn’t match.” (Participant #104, physiotherapist with 13 years’ experience) “We have parents who read [the record] all the time, they read every little note the nurses make. And they can look at the time of a note, correct spelling errors or… And you feel very, very observed, and I’m not sure that it leads to doing a better job, instead you spend time thinking about exactly how to phrase things than you might otherwise do.” (Participant #18, physician with 12 years’ experience)
Threatened confidentiality	“One can very well imagine that parents more or less force themselves into the record through the teenagers.” [Participant #41, physician with 12 years’ experience] “The child might not dare to speak freely with a psychologist or with doctors or other HCPs about things they don’t want their parents to read. So it inhibits the children in that way.” (Participant #104, physiotherapist with 13 years’ experience)
Technical portal limitations	“I had such a misunderstanding just yesterday where I wrote that they should do a kidney examination (“njurundersökning”), and autocorrect wrote ‘pleasure examination’ (“njutningsundersökning”).” (Participant #118, nurse with 13 years’ experience)

Most HCPs described that ORA might lead to an *increased emotional distress and confusion* among adolescents and parents, such as from not understanding results or the healthcare processes, causing them to worry. One participant described that the simplicity of the information in the EHR may worry parents, who are unaware of decision-making processes involving numerous HCPs and extensive discussion. Several HCPs referred to the “life and death” nature of news communicated to oncology patients as a reason for particular concern in communicating news to adolescents and parents before having the opportunity to explain it. Furthermore, one HCP described a situation where a divorced parent had become distrustful of the other due to information about them related to alcohol abuse, that they could read in the child’s EHR. Some HCPs mentioned that sole access for adolescents may lead to difficult family situations, where the adolescent may not inform the parent about concerning information because of not wanting to worry them, as reported by this physician: “Perhaps [teens] don’t want to tell their parents or siblings what they have read because they become worried themselves and don’t want to worry their parents.” (Participant #72, physician with 12 years’ experience).

Partly due to concerns about causing distress, participants reported that ORA contributed to a *decreased documentation quality and accuracy*. HCPs described having to be cautious when writing and being vaguer, which could lead to potential misunderstandings with other HCPs. HCPs mentioned having to omit information, such as sensitive information about either the child or the parent, or hypotheses related to the illness. Physicians reported that omitted information would sometimes be communicated outside of the record, which was cited as a patient safety risk. In some cases, HCPs would wait to release information due to concern of worrying parents, which also led to delay of that information reaching other HCPs, as this physician stated: “I know that sometimes you can ponder a bit about that, and that some hesitate a bit to… to write before they’ve had a chance to talk, which means that colleagues and… who could potentially benefit from this, get it later as well.” (Participant #116, physician with 38 years’ experience).

Several participants experienced that ORA caused an *increased workload*, as worried parents would call in and ask about information they did not understand. Furthermore, HCPs noted that some parents would read the records compulsively and even during the visit, which affected HCPs’ work environment negatively. While finding this behavior understandable and stating that it was not very common, HCPs found it stressful. Lastly, a few HCPs mentioned the risk of threat to staff: “Because these illnesses are so severe, [the parents] are so scared and stressed that they can become angry and aggressive, and even threatening. It doesn’t happen very often, but regularly still. It can be tough.” (Participant #72, physician with 12 years’ experience).

Participants worried that ORA *threatened the confidentiality* of adolescents and parents. For example, when adolescents had access, parents could access their child’s account by coercion. Some recounted experienced cases of controlling parents reading their adolescent girls’ EHR without consent. Despite that information perceived as sensitive was not usually directly related to cancer treatment, adolescents with cancer may have questions about sex and alcohol which affect or be affected by the treatment. HCPs held that adolescents may conceal information or refrain from seeking healthcare if worried about their parents accessing the records. Also, some HCPs stated a concern in that adolescents would share information with friends or on social media: “There isn’t much consideration for the consequences of the information in the EHR, if it’s spread, what consequences it might have. One might take screenshots of the EHR and post them on social media and things like that.” (Participant #02, nurse with 23 years’ experience).

Some HCPs described *technical portal limitations* that created issues. For example, they perceived the available hidden search words to not sufficiently cover the topics that cause a need for concealment. Also, that differences in information availability between regions created inequality for patients.

#### Perceptions of adolescent and parental regulations

Five themes were identified with respect to participants’ views on regulations: *Uncertainty*, *Adolescents differ*, *Balancing parental support and adolescent privacy*, *“I understand why access is restricted*,* but…”*, and *Regulatory changes* (see Table 5).

**Table 5:** Themes of hcps’ views on regulations for adolescent and parental ORA

Themes	Sub-theme	Representative quotes
Uncertainty	Regulations	“I’m still a little, little hesitant [about how it works]. That’s why I thought, I’ll participate in the interview and learn from you.” (Participant #22, nurse with 34 years’ experience) ”I think the parents are quite aware [that they will lose their access]. However, the healthcare system does not always know about it. They have no idea that parents do not have access to the medical record” (Participant #03, nurse with 22 years’ experience)
	Extension	“Parents ask: ‘can I get authorization to access my child’s medical record?’ […] It has probably come up a couple of times. And then I have talked to the secretaries, who I think … Now I feel uncertain. I think they have said that otherwise you can request the medical record to be printed, because you can at least do that, right?” (Participant #118, nurse with 13 years’ experience)
	Adolescent and parental access and use	“If parents receive an X-ray result before we do, or if children don’t want us to write things in the medical record because they don’t want their mom and dad to know, it’s so darn difficult to know what is shown when you block a record, and what shows when using different search terms.” (Participant #18, physician with 12 years’ experience)
Adolescents differ	Low perceived use and interest	“My experience is that [adolescents in general] come here, lie down in bed, get their treatment, and then go home when they’re done. They don’t want to know, and then it’s difficult if they are the ones who should get the information to then pass on, and keep track of ‘how long should I take these medicines at home?‘” (Participant #02, nurse with 23 years’ experience) “I think the times they log in, that we hear about them logging in, it’s more at the parents’ request to check’ what time we’re supposed to be there’ or ‘how your tests look’, and so on. That maybe it’s not so much their own need they’re logging in for.” (Participant #33, nurse with 13 years’ experience)
	Depends on factors other than age	“You’re not very mature when you’re 13 years old. You don’t have much responsibility yourself. But at the same time, some mature much, much earlier than others. So, it’s incredibly difficult to pinpoint an exact age.” (Participant #22, nurse with 34 years’ experience) “It varies greatly depending on one’s level of interest and developmental stage. But I think having the option available can be really beneficial.” (Participant #22, nurse with 34 years’ experience)
	Involvement	“There are some teenagers who keep track of their test results and manage a lot of that themselves. Even if parents are involved too, they are still actively participating in their own care.” (Participant #06, nurse with 1 year of experience)
Balancing parental support and adolescent privacy	Sick adolescents depend on parents	“Even a 17-year-old or a 17.5-year-old who is newly diagnosed almost always becomes dependent on their parent and needs their parent. Then it feels more like they are transferring the responsibility to the parents.” (Participant #03, nurse with 22 years’ experience) “We work with the whole family, and I still haven’t encountered any teenager who says ‘Mom and Dad aren’t allowed to see what I’m doing in cancer treatment,’ and then one might need to be there to support one’s teenager. Because it’s tough if it’s only the teenager who has access to those parts.” (Participant #22, nurse with 34 years’ experience)
	Allowing privacy	“Some say ‘yeah, I don’t care either way, you can check.’ And some say like ‘no, that’s mine. Why are you snooping?’ So I think it’s very different, and people have very different relationships with their parents.” (Participant #118, nurse with 13 years’ experience) “A 13-year-old, a teenager, is already quite vulnerable. If, for example, you are going to some kind of youth clinic or something similar where records are kept anyways, I still think it’s somehow positive that you can maintain some anonymity from your parents in that way.” (Participant #81, nurse with 5 years’ experience)
	Controlling parents	“For most parents, they could potentially have access for a little longer, and for the parents who can’t have it between 13 and 16, maybe they shouldn’t have it before 13 either.” (Participant #18, physician with 12 years’ experience)
“I understand why access is restricted, but…”	Hinders care	“I think it’s a scandal to be honest. Because it gets incredibly difficult to manage for our families. It depends a little. Those who are undergoing treatment, it gets really hard. Because the parents can’t see and they need to call regarding lab results. They already have their hands full and it gets hard for them.” [Participant #03, nurse with 22 years’ experience] “It’s also very strange because then you think, what is it between 13 and 16 that makes it sacred so that no one can access it? I mean, if you have a child who gets sick at 12 years and 9 months, then the parents will still want to have an understanding of what’s going on. And what is it that makes it locked during that period? And how does it benefit healthcare that it’s closed?” (Participant #118, nurse with 13 years’ experience)
	Losing access is frustrating for parents	“It’s often frustrating when they can’t access it themselves, because they usually check test results and such. Then they have to ask us instead, and… […] when they’re at home, they might not want to call. So, it’s often tough when they don’t have access.” (Participant #06, nurse with 1 year of experience) “Some [parents] ask immediately when it happens: ‘why can’t I see this or that?’ […] It depends a bit on age; some who are around 14–15 usually have a good handle on things, but those in the middle, around 11–12, transitioning to 13, might be more shocked and perhaps irritated instead.” (Participant #81, nurse with 5 years’ experience)
	Cumbersome application process	“Since we have a shortage of places, we relocate the children to other regions, and then the parents need to apply for access there as well. […] After a while, parents need to fill in four, five forms. And on top of that one has regular work to do, and maybe more children to take care of.” [Participant #02, nurse with 23 years’ experience]
Regulatory changes	Close access gap	“I think there could be shared responsibility between 13 and 16.” (Participant #03, nurse with 22 years’ experience) “I understand the idea that maybe you’re not old enough to understand, but if no one… then I think you should have access when you’re 14, 15 in that case, when parents no longer have access.” (Participant #06, nurse with 1 year of experience)
	Enhance information and process for extended access	“I just find it strange that there isn’t more outreach… that there aren’t informative brochures available at the hospital, for example. Something like, ‘Did you know you can access your medical records?’” (Participant #104, physiotherapist with 13 years’ experience) “In pediatric oncology, it should be possible for the head of the home hospital to approve access to the pediatric oncology record at all children’s hospitals. […] And now it’s a paper form to fill out, and I’m thinking, can we look into digital solutions for the whole process, where parents apply digitally? And then the head of the department approves or rejects it digitally as well.” (Participant #02, nurse with 23 years’ experience)
	Customized record	“I think it would be easier if we could control which things everyone would have access to, rather than controlling different ages and so on. Because then it might get a bit messy to explain as well, like ‘no, you’re not a mature 13-year-old, you can’t have this.‘” (Participant #22, nurse with 34 years’ experience) “Sometimes one would wish that there were… well, there are, but… it might be utopian, but that there were different records. One directed towards… between healthcare providers and one directed towards families.” (Participant #03, nurse with 22 years’ experience)

Many participants described *uncertainty* regarding regulations, extension and record availability and ORA use among adolescents and parents. While largely aware of the age limits, HCPs appeared less well-informed about the possibility of access extensions (especially regarding adolescents) and the application procedure, such as this physiotherapist: “I initially thought that it was only the parents who could apply for permission to read. I was actually unaware that the children could do it.” (Participant #104, physiotherapist with 13 years’ experience). One HCP mentioned that when informed about the policy, the general idea had been to learn details when necessary. While most had not experienced an application for extension, almost all who had experienced it described the application procedure as challenging, and only one participant had personal experience of attempting to apply, and subsequently gave up.

Participants described that *adolescents differ*. They stated that amongst adolescents with cancer, most did not appear to have any interest in reading their records and preferred to depend on their parents. Adolescents rarely asked questions and preferred asking via parents; “Let’s say a 10-, 12-year-old with cancer is still quite dependent on their parents and… no, it may be bad, but it feels like they are quite content… I mean they are fighting to survive, they don’t have much interest in reading their records.” [Participant #03, nurse with 22 years’ experience]. Exceptions did exist, for example mature adolescents with an interest in tracking their test results. HCPs emphasized that type of health issue likely led to different needs for adolescents to read their records. Some imagined that ORA could provide benefits for adolescents who may be especially receptive due to their digital competence, and suggested informing adolescents more about the EHR: “Since it’s a natural part of growing up to have your phone or iPad or… the EHR somehow becomes more accessible.” (Participant #118, nurse with 13 years’ experience).

Another emergent theme in relation to regulations was *balancing parental support and adolescent privacy*. HCPs stated that adolescents have varying needs for privacy, and that ill adolescents “tend to regress” during the illness period and depend on parental support, regardless of age. For these adolescents, it was primarily parents who wanted ORA. Meanwhile, some HCPs were concerned about infringements on the privacy of adolescents with cancer, given parents’ (understandable) concern and interest in their information. A number of HCPs noted that it did not matter whether only the adolescents had access because parents would often ask the adolescent to log in for them to read. In regards to extended access, HCPs held that it should require child consent and be updated every six months/year, as situations can change between age 13–15.

“That older teenagers need to log in because their parents absolutely want to know, we’ve seen that.” (Participant #41, physician with 12 years’ experience).

A fourth theme was *“I understand why access is restricted*,* but…”*. HCPs expressed understanding the need for restricting access, but they were still confused and critical in regards to the gap in access between age 13–15, especially in regards to families of adolescents with cancer. Upon losing access to their child’s records at age 13, HCPs perceived that many parents were confused, frustrated and shocked. Several HCPs reported a need to better inform parents prior to this event. Furthermore, adolescents started receiving notifications via the patient portal at age 16, which was unknown to patients and led to patients missing appointments. Access extension was described as difficult for a number of reasons, such as that parents of children with cancer live in chaos and struggle to submit one application for each unit, which can be many.

“I tell [parents] that it’s not something you fix in fifteen minutes. Because you don’t. It takes weeks, as it has in the cases I’ve been involved in.” [Participant #03, nurse with 22 years’ experience].

Lastly, HCPs’ provided a number of suggestions for *regulatory change* that they perceived would improve ORA for adolescents battling cancer and their parents. Several suggestions from HCPs concerned facilitation of the extension application. More information was needed for parents, and support for digital applications and approval. It was reported that permissions for extended access need renewal every six months or annually, and that the child should be consulted. Considering the significance of life events for ORA needs, some were skeptical of an age-based system. One HCP mentioned that separate records for HCPs and adolescents/parents may prevent issues related to documentation issues; “Sometimes one would wish that there were… it might be utopian, but that there were different records. One directed towards… between healthcare providers and one directed towards families.” [Participant #03, nurse with 22 years’ experience].

## Discussion

### Summary of findings

The study provides insight into pediatric oncology HCPs’ perspectives of ORA for parents and adolescents in Sweden. Our survey and interview findings demonstrate that most HCPs differed in their views on regulations, but were critical of restricted access between age 13–15 for both parents and adolescents. HCPs see potential in adolescent ORA but do not perceive high use among adolescent with cancer, who tend to depend on parents. While positive about ORA improving parents’ ability to support their child, HCPs were concerned about the quality of the EHR when documenting with consideration of adolescents’ and parents’ privacy. HCPs lack information about regulations to some degree, but mainly about procedures to extend access and what is shown to users.

### Comparison with prior work

This is the first study to examine pediatric HCPs’ perceived benefits and risks of adolescents’ and parents’ ORA, as well as views of a regulatory framework for pediatric EHRs.

A larger proportion of HCPs were positive to parents’ access for children under 13 and the utility of parental access in their work, compared with adolescents’ access. A possible explanation for this is that adolescents with cancer did not appear to be reading their records, thus their access did not affect HCPs. The perception of adolescents as rare users is aligned with a previous US study on pediatric oncology clinicians [8] and might be accurate, given that provider encouragement among adolescents is commonly low [2], they often lack knowledge about patient portals [30] and value normalcy when seriously ill [31]. Another possible factor could be that adolescents prefer to ask questions via parents [32] and may lack confidence in clinical environments [33]. Also, HCPs lacked knowledge about information visibility in the EHR and whether parents and adolescents were reading the records, which prevented using the EHR as a tool of communication. Previous work has identified that compared with other HCPs, a minority of oncologists see ORA as a means for communication [9]. In general, perceived benefits of access often concerned anticipatory effects (such as ability to correct information or view test results), while several concerns were experienced by the HCPs, such as modifying documentation or being confronted by worried or confused parents. Thus, the benefits did not affect the HCPs positively, in the way that concerns affected them negatively. This imbalance, where risks are experienced and tangible but benefits remain hypothetical, may contribute to low ratings of utility and amotivation to encourage patients to use PAEHRs [34, 35].

Despite low perceptions of utility from adolescents’ and parental ORA, HCPs were critical to the access restrictions. The complications of restricting ORA for parents of seriously ill children has been noted in the literature [8, 16]. Possibly due to HCPs’ advocacy for adolescents’ autonomy and privacy, slightly more HCPs were positive about adolescents’ being able to gain earlier access than parents’ prolonged access. Threats to confidentiality related to parental ORA was often described as a reason for HCPs’ difficulties to document sensitive information and subsequent need to omit information from the record. Some HCPs suggested shared access during adolescence, a solution that has been observed as leading to confidentiality concerns. However, attempts have been made at innovative portal functionality, where sensitive information is stored separately and only visible to the adolescent [36]. Compromised note quality has been previously anticipated in pediatric care [37] and selective omission within the records to ensure confidentiality has been observed among pediatric providers [13]. Furthermore, a survey study found that oncologists worry more than non-oncology HCPs about decreased quality of documentation [9], possibly due to the serious nature of some information. Suggestions from participants included allowing HCPs to choose which aspects of the record should be available to adolescents and parents, as proposed in mental healthcare [38], and creating dual records for HCPs and families. Although these strategies might reduce harm or misunderstandings, such solutions carry significant ethical and practical challenges including concerns about transparency, increased documentation burden, and the risk of undermining trust if patients sense selective disclosure. In their struggle to set age limits, HCPs suggested that access should instead be based on type of care and HCPs should have larger control of what is shown. Other countries have adopted case-by-case approaches, for example, HCPs in Finland assess minors’ decision-making capacity for each care event, and allow those able to consent to parental view of the information [39]. While allowing larger flexibility, subjective assessments can lead to patient inequality and increased HCP work burden, requiring guidance and resources.

HCPs’ lack of knowledge about the application procedure for extended access is notable, as it likely poses a key barrier to adolescent and parental use of ORA during adolescence. In line with this finding, a previous case study identified very low numbers of applications, disproportionate to population size, in the five of Sweden’s 21 regions [39]. Furthermore, differences in knowledge among HCPs may lead to unequally distributed opportunities of ORA for adolescents and parents. The study also found that early access can lead to higher engagement during late adolescence, which is again prevented by a lack of HCP knowledge. The perceived potential for adolescents of ORA led a few HCPs to suggest a need to better inform adolescents about the possibility of reading their records. As this was primarily suggested by nurses, future work is needed to explore differences in views between physicians and nurses. Overall, the findings highlight the tension between HCPs’ need for professional autonomy in EHR documentation, and a transparency that is central to patient empowerment. ORA may be perceived as constraining communication between HCPs, particularly in the clinically complex context of pediatric oncology care - raising questions about how to balance openness with professional discretion required for safe and effective care.

Another concern of HCPs was related to impact on work and documentation, including compromised clinician autonomy, a concern that has been raised in the literature [1]. For example, concerns about confidentiality and rapid release of results have led HCPs to modify their documentation by omitting information, delaying its release, and communicating externally from the EHR. In a previous study, oncology clinicians stated additional strategies to prevent potential harm, such as establishing agreements with radiologists and providing families with anticipatory guidance [8]. Adjustments such as omitting information from the EHR could lead to risks for patient safety, which was also noted by HCPs in this study, and may even incur medical-legal challenges [40]. In accordance with prior findings, HCPs also mentioned technological limitations in concealing sensitive information [41]. Overall, these findings call for portal design that allow concealment of sensitive information from parents and guidance for HCPs to release information while decreasing harm to families. Supporting HCPs in using ORA is critical in order to relieve their concerns and allow for integrating patient portals into care, as a tool to improve partnerships between HCPs, parents, and adolescents. Another emerging opportunity is the use of artificial intelligence (AI) tools to support clinical documentation, such as ambient scribing. While such tools may facilitate the administrative burden on HCPs, they also raise concerns around patient privacy and the risk of documentation errors, warranting further research.

### Implications

Based on the findings, we have outlined key implications for both practice and research.

Implications for practice:

- Short-term:
  - Provide HCPs with information about PAEHRs, including use, updates, privacy functionality, release expectations, information visibility for children, adolescents, and parents.
  - Provide HCPs with information on regulations related to extended access, and guidance on how to facilitate the application procedure for adolescents and parents.
  - Provide adolescents and parents with clear, accessible written information about ORA (e.g. on the national patient portal and via brochures in clinics and hospitals).
- Long-term:
  - Facilitate the extended access application process by digitizing application forms and enabling adolescents and parents to complete and submit them online.
  - Train HCPs on writing clinical notes that are accessible to patients, including adolescents and parents.
  - Foster dialogue between oncology HCPs, adolescents, and parents regarding ORA, including note sharing, communication, timing of release and concealment of sensitive information.
  - Develop functionality that enables HCPs to see whether adolescent patients or parents have viewed a specific note.

Implications for research:

- Explore the potential of generative artificial intelligence tools in supporting HCPs with clinical documentation.
- Investigate differences in perspectives on adolescents and proxy ORA between physicians and nurses, as well as across Swedish regions.
- Examine how HCP training in writing EHRs and increased patient guidance can reduce anxiety in minors and parents when reading EHRs.
- Assess changes in documentation practices due to ORA.

### Strengths and limitations

This is the first study to explore pediatric oncology HCPs’ views and awareness of regulations for adolescent and parental ORA, and the findings of two collection methods used reflected each other. Yet, the study is not without limitations. First, the study sample was small and survey respondents represented only 8 of 21 Swedish regions, which restricts the ability to generalize findings to all HCPs and may not capture regional variations. Future research should aim to include all regions, allowing for exploration of views and experiences across regions. Responder bias may also have affected findings: for example, it is possible that those who chose to respond and subsequently participate in an interview were more enthusiastic about ORA, or more skeptical HCPs participated. Participants may also have been impacted by recall bias. Most participants were women and nurses, yet the sample may still be representative since in Sweden, most nurses are women and nursing is the largest healthcare profession [42]. The survey used was designed by the authors due to a lack of validated questionnaires available for examining views on ORA, however it was piloted with pediatric oncology HCPs for feedback prior to study start. Another limitation is that we did not examine adolescents’ and parents’ views and experiences. This was however the aim of a previous study conducted in parallel that is now published [43]. An interview guide was followed to improve trustworthiness, and the study was reported according to the COREQ checklist. Using a qualitative approach, researcher reflexivity is vital to mitigate bias. For example, multiple researchers were involved in the coding process, and differences in interpretation were resolved through dialogue and consensus. Also, the research team included members with clinical and qualitative research experience.

## Conclusion

In this study, most pediatric oncology HCPs in Sweden were critical about ORA restrictions among adolescent oncology patients. In contrast, they perceived benefits of ORA primarily with respect to parental access. In addition, the importance of strengthening adolescents’ autonomy was also emphasized by participants. However, most HCPs lacked knowledge about exemptions, information availability and use among adolescents and parents. Uncertainty, along with confidentiality concerns, contributed to HCPs’ perception that documentation quality was compromised. Better understanding about the patient use and integration of ORA within healthcare was requested. Guidance in mitigating anticipated harm may increase HCPs’ perceived utility of patient portals for adolescents and parents. Furthermore, increased flexibility of regulation, facilitation of procedures and functionality to ensure confidentiality is necessary to ensure patient safety and quality of documentation.

## Supplementary Information

Below is the link to the electronic supplementary material.

Supplementary Material 1

Supplementary Material 2

Supplementary Material 3

Supplementary Material 4

## Data Availability

The study data are not publicly available due to concerns about confidentiality of a small sample. Quotations and analytical categories are included in the text. Contact the corresponding author to discuss the findings or the analysis. Redacted versions of data are available upon reasonable request.

## Abbreviations

COREQ: Consolidated criteria for Reporting Qualitative research
EHR: Electronic health record
EU: European Union
GDPR: General Data Protection Regulation
HCP: Healthcare professional
ORA: Online record access
PAEHR: Patient-accessible electronic health record
US: United States
