# Supplementary material for: Pediatric oncology healthcare professionals’ attitudes to and awareness of regulations for minors’ and guardians’ online record access: a mixed-methods study in Sweden
Source: BMC Health Serv Res. 2025 Nov 27;25:1562. doi: 10.1186/s12913-025-13697-3 (PMC12670769; doi:10.1186/s12913-025-13697-3)
Supplement: Supplementary file 1 — Supplementary Material 1 [file 12913_2025_13697_MOESM1_ESM.pdf]

Supplementary Material 1

Consolidated criteria for reporting qualitative studies (COREQ): 32-item checklist.

No	Item	Guide questions/description	Remarks	Section, subsection
**Domain 1: Research team and reflexivity**				
Personal Characteristics				
1.	Interviewer/ facilitator	Which author/s conducted the interview or focus group?	Main author JH led all interviews and focus groups	Methods, data collection
2.	Credentials	What were the researcher's credentials? *E.g. PhD, MD*	JH: MSc, psychology. MH: PhD.	Methods
3.	Occupation	What was their occupation at the time of the study?	JH: PhD candidate	Methods, data collection.
4.	Gender	Was the researcher male or female?	JH: female	Methods, data collection
5.	Experience and training	What experience or training did the researcher have?	JH has undergone courses in qualitative research and has previous experience with qualitative research, and analyzing data with NVivo.	Methods, research team and reflexivity
Relationship with participants				
6.	Relationship established	Was a relationship established prior to study commencement?	JH had no professional-client relationship with any of the participants.	Methods, research team and reflexivity
7.	Participant knowledge of the interviewer	What did the participants know about the researcher? *e.g. personal goals, reasons for doing the research*	At the start of each interview, JH introduced herself and the reasons for doing the research.	Methods, research team and reflexivity
8.	Interviewer characteristi cs	What characteristics were reported about the interviewer/facilitator? *e.g. Bias, assumptions, reasons and interests in the research topic*	PhD student	Methods, discussion.
**Domain 2: study design**				
Theoretical framework				
9.	Methodolog ical orientation and theory	What methodological orientation was stated to underpin the study? *e.g. grounded theory, discourse analysis, ethnography, phenomenology, content analysis.*	A thematic, inductive content analysis was conducted.	Methods, research design
Participant selection				
10.	Sampling	How were participants selected? *e.g. purposive, convenience, consecutive, snowball*	Purposive sampling, aiming for a presentation of both sexes, parents and adolescents, different educational levels, both native and migrant background, coming from all participating municipalities and visitors of both preventive health care and youth and social care.	Methods, study population and inclusion.
11.	Method of approach	How were participants approached? *e.g. face-to-face, telephone, mail, email*	E-mail or face-to-face invitation by a professional, followed by phone/email from one of the researchers to make appointment.	Methods, study population and inclusion.
12.	Sample size	How many participants were in the study?	13 participants participated in interviews.	Results, general characteristics. Table 1: 'characteristics participants'
13.	Non-particip ation	How many people refused to participate or dropped out? Reasons?	6 of 19 HCPs who registered interest in the survey did not participate, due to scheduling difficulties.	Results
Setting				
14.	Setting of data collection	Where was the data collected? *e.g. home, clinic, workplace*	One interview was conducted via phone and 12 were conducted via	Methods

			the video-conferencing software Zoom.	
15.	Presence of non-particip ants	Was anyone else present besides the participants and researchers?	No	n/a
16.	Description of sample	What are the important characteristics of the sample? e.*g. demographic data, date*	Demographics (gender, age, profession, years of professional experience), and interview setting have been represented in table 2	Results (Participant demographic characteristics) table 1-2
Data collection				
17.	Interview guide	Were questions, prompts, guides provided by the authors? Was it pilot tested?	An interview guide was written by the authors and tested with HCPs. The guide was slightly revised based on their suggestions.	Methods, data collection
18.	Repeat interviews	Were repeat interviews carried out? If yes, how many?	No	n/a
19.	Audio/visua l recording	Did the research use audio or visual recording to collect the data?	All interviews were audio-recorded. The visual recordings for video calls were not stored.	Methods, data collection
20.	Field notes	Were field notes made during and/or after the interview or focus group?	No	n/a
21.	Duration	What was the duration of the interviews or focus group?	Individual interviews ranged from 20-79 minutes (mean 44 minutes).	Methods, data collection
22.	Data saturation	Was data saturation discussed?	Yes	Methods, data collection
23.	Transcripts returned	Were transcripts returned to participants for comment and/or correction?	No	n/a
**Domain 3: analysis and findings**				
Data analysis				
24.	Number of data coders	How many data coders coded the data?	Two authors (JH and MH)	Methods, data analysis
25.	Description of the coding tree	Did authors provide a description of the coding tree?	No	n/a
26.	Derivation of themes	Were themes identified in advance or derived from the data?	Themes were derived from the data. Benefits and risks were inspired by prior work.	Methods, data analysis
27.	Software	What software, if applicable, was used to manage the data?	NVivo v.1.7.2	Methods, data analysis
28.	Participant checking	Did participants provide feedback on the findings?	No	na
Reporting				
29.	Quotations presented	Were participant quotations presented to illustrate the themes / findings? Was each quotation identified? *e.g., participant numbe*r	Yes, identified by profession, years of experience, and participant number. The participant number is indicated as # followed by the number of the interview.	Results
30.	Data and findings consistent	Was there consistency between the data presented and the findings?	Yes	Results

31.	Clarity of major themes	Were major themes clearly presented in the findings?	Yes	Results
32.	Clarity of minor themes	Is there a description of diverse cases or discussion of minor themes?	Yes	Results

Allison Tong, Peter Sainsbury, Jonathan Craig, Consolidated criteria for reporting qualitative research (COREQ): a 32-item checklist for interviews and focus groups, International Journal for Quality in Health Care, Volume 19, Issue 6, December 2007, Pages 349–357, DOI 10.1093/intqhc/mzm042
