# Supplementary material for: Pediatric oncology healthcare professionals’ attitudes to and awareness of regulations for minors’ and guardians’ online record access: a mixed-methods study in Sweden
Source: BMC Health Serv Res. 2025 Nov 27;25:1562. doi: 10.1186/s12913-025-13697-3 (PMC12670769; doi:10.1186/s12913-025-13697-3)
Supplement: Supplementary file 2 — Supplementary Material 2 [file 12913_2025_13697_MOESM2_ESM.pdf]

Appendix 3

Interview guide for healthcare professionals

Pediatric oncology

I. Introduction

1 Hi ____ and thank you for wanting to participate in this interview. My name is [First and last name], I am a [title] at [institution] and working on this project.
2 In this interview, I would like to ask about your experiences in your work about how adolescents who have been treated for cancer, and their parents have access to and use medical records online on [name of EHR service].
3 Everything you say will be treated confidentially and you are anonymous.
4 During the interview, you may think of something I do not ask about. There are no right or wrong answers to my questions, so just answer as you feel it is.
5 Do you have a question before we start?

II. Background questions

We will start with some background questions.

1 How old are you?
2 How long have you worked in pediatric oncology?
3 Did you work in healthcare when records became available on the internet?
  a If so, what were your thoughts on it then?

III. Questions about the EHR regulations

Now I will ask about your thoughts on the regulations for the records for adolescents.

1 Do you know what the regulations look like for parents' and children's access to the record?
  a What do you think about it?
2 The parent loses access to their child's medical record at the age of 13.
  a Do you feel that parents know that they will lose their access when the child turns 13?
  b How do you feel about parents reacting to losing access to their child's records?
  c Does it affect you in your work in any way? (Complicating/facilitating) [Is 13 years old an appropriate age? Do you think parents should have longer access? Why?]
3 Young people get access to their medical records when they turn 16.
  a Do you think this is an appropriate age?
  b Do you feel that many adolescents are interested in reading their records?
4 Between 13 and 16 years old, neither parents nor children have access to the child's records.
  a Does this affect your work? In what way?

IV. Extended access to EHR

During the period when the child is 13-15, it is possible for parents and adolescents to apply for extended access to the records. Now I will ask about your thoughts on it.

5 Do you know that it is possible for parents to apply for extended access?
  a If so, how did you get this information? Do you know what the application process looks like?
  b Do you usually inform parents about the possibility of extending their access to the medical record during the child's visit?

    i. If so, why and how?
    ii. If not, why?

  c Have you ever received an application for extended access to the child's records for a parent?
    i. If yes, was the application approved / denied? Why? How did the applicant react?
  d Have you at any time removed a parent's access to their child's records?

6 Are you aware that it is possible for adolescents to apply for access earlier than at the age of 16?
  a If so, how did you get this information? Do you know what the application process looks like?
  b Do you usually inform adolescents about the possibility of applying for access? Why/why not?
  c Have you ever received an application for earlier access to records for a child under the age of 16 who was treated for cancer?
    i. If yes, was the application approved / denied? Why? How did the applicant react?
    ii. Have you encountered any youth who already had extended access?

V. Parents' access to their child's medical records

Now I will ask about your thoughts on parents' access to their child's records.

7 What do you see as the benefits of parents' access to their child's records online?
  a What are the disadvantages? Feel free to describe examples.
8 Is anything in the child's records ever discussed with the parent during a visit?
9 Do you inform parents about records online during the child's visit?
10 Do you feel that it is common for parents to use the records in the care of their child? If yes, how?
11 Do you feel that parents know that they have access to their child's medical record online?

VI. Adolescents' access to their EHR

Now I will ask about your thoughts on adolescents' access to the records.

12 What are the benefits of adolescents having access to their records?
  a What disadvantages do you see? Feel free to describe examples.
13 Do you experience/think that adolescents who are treated for cancer and their parents use the records in a different way than healthy adolescents?
14 Does it affect your work that adolescents have access to their records over the internet when they turn 16? How?
15 Do you get questions from adolescents about things that are read in their records?
  a If so, what can such questions be about?
16 Do you adapt your way of writing when you know that it concerns an adolescent who can then read your note?
  a If so, in what way are you changing your way of writing?
  b Does it affect your way of documenting / managing the records in any other way?

VII. Additional comments

1 Do you have any more comments or something you are thinking of that we have not talked about?

2 Do you have any comments on why you chose to participate?

VIII. Conclusion

1 Now we have reached the end of the interview.

2 We will now continue to talk to parents, adolescents and care staff. If or when the research is eventually published, information will be spread on these web pages (state where) so there it may appear.
Do you want us to save your e-mail address for information when a study is published? YES/NO
Thanks again for taking the time to participate.
