# Supplementary material for: Pediatric oncology healthcare professionals’ attitudes to and awareness of regulations for minors’ and guardians’ online record access: a mixed-methods study in Sweden
Source: BMC Health Serv Res. 2025 Nov 27;25:1562. doi: 10.1186/s12913-025-13697-3 (PMC12670769; doi:10.1186/s12913-025-13697-3)
Supplement: Supplementary file 3 — Supplementary Material 3 [file 12913_2025_13697_MOESM3_ESM.pdf]

Appendix 3

Results

Perceptions of parental and adolescent ORA regulations.
1=Completely disagree, 5=Completely agree.

Statement, n (%)	1	2	3	4	5	Don't know	Missing
For children under 13, it is a good principle that parents have access to their child's EHR	2 (2.1)	7 (7.4)	5 (5.3)	24 (25.3)	57 (60)	0	0
It is generally good that adolescents have access to their EHR	1 (1.1)	10 (10.8)	19 (20.4)	28 (30.1)	35 (37.6)	1	1
It is good that no one (parents or children) has access to the EHR when the child is 13-15 years old	40 (44.4)	25 (27.8)	16 (17)8	7 (7.8)	2 (2.2)	4	1
It is good that the parent can apply for extended access for the parent	3 (3.4)	8 (9.0)	12 (13.5)	20 (22.5)	46 (51.7)	6	0
It is good that young people between 13-15 can apply for extended access	3 (3.6)	3 (3.6)	13 (15.7)	24 (28.9)	40 (48.2)	12	0
In my work, it is helpful if the parent has access to their child's EHR	15 (16.5)	11 (12.1)	16 (17.6)	19 (20.9)	30 (33.0)	4	0
In my work, it is helpful if children have access to their EHR	18 (18.9)	15 (17.2)	25 (28.2)	10 (10.5)	19 (20.0)	7	1

Note: Percentages were calculated by excluding missing data and "don't know" responses.
